# A novel model for percentile-based oscillometric blood pressure reference values in Danish children: implications for the use of international standards in pediatric screening

**DOI:** 10.1007/s00431-026-06969-5

**Published:** 2026-04-30

**Authors:** Lise Fischer Mikkelsen, Henrik Enghusen Poulsen, Mikkel Porsborg Andersen, Søren Hagstrøm, Konstantinos Kamperis, Jørgen Kim Kanters, Christina Ellervik, Luise Borch

**Affiliations:** 1 Department of Pediatric and Adolescent Medicine, Gødstrup Hospital, Herning, Gødstrup Denmark; 2 Department of Clinical Medicine, Aarhus University, Aarhus, Denmark; 3 Department of Endocrinology, Bispebjerg Frederiksberg University Hospital, Copenhagen, Denmark; 4 Copenhagen University Hospital–Steno Diabetes Centre Copenhagen, Herlev, Denmark; 5 Prehospital Centre, Region Zealand, Næstved, Denmark; 6 Department of Health Science and Technology, Aalborg University, Aalborg, Denmark; 7 Department of Clinical Medicine, Aalborg University, Aalborg, Denmark; 8 Department of Pediatric and Adolescent Medicine, Aalborg University Hospital, Aalborg, Denmark; 9 Department of Pediatrics and Adolescent Medicine, Aarhus University Hospital, Aarhus, Denmark; 10 Laboratory of Experimental Cardiology, Department of Biomedical Sciences, University of Copenhagen, Copenhagen, Denmark; 11 Center of Biosignal Research, University of California San Francisco, San Francisco, USA; 12 Department of Clinical Biochemistry, Zealand University Hospital, Køge, Denmark; 13 Department of Clinical Medicine, Faculty of Health and Medical Sciences, University of Copenhagen, Copenhagen, Denmark; 14 Department of Laboratory Medicine, Boston Children’s Hospital, Boston, USA; 15 Department of Pathology, Harvard Medical School, Boston, USA

**Keywords:** Hypertension, Oscillometric reference values, Blood pressure, Pediatric

## Abstract

Accurate blood pressure reference values are crucial for detecting hypertension in children. However, international guidelines, with reference values based on multiethnic US cohorts, may not be fully applicable in other populations. This study primarily aimed to develop percentile-based population-specific oscillometric blood pressure reference values for Danish children and, secondly, to compare these to existing percentile-based international reference values. A cross-sectional study was conducted using data from the Lolland–Falster Health Study (2016–2020), including 1771 children aged 4–15 years. A reference model was developed based on 1512 children of normal weight, excluding those with chronic conditions, blood pressure-altering medications, or a country of origin other than Denmark. Quantile regression models estimated sex- and age-specific 5th to 95th percentile for systolic and diastolic blood pressure. Reference values were compared in reclassification tables. The novel Danish reference model provides percentile-based age- and sex-specific oscillometric office blood pressure reference values. Excluding height from the model had minimal impact on explanatory power. Applying international reference values identified 27% to 69% of the Danish children with blood pressure at or above the 95th percentile, when using the novel Danish reference values as an internal comparative benchmark.

*Conclusion*: This study provided percentile-based reference values for oscillometric office blood pressure screening in children and adolescents of Danish origin and demonstrated that applying international reference values across different pediatric populations and measurement modalities may carry risk of misclassification. These findings suggest a need for population-tailored reference values in improving the diagnosis and management of hypertension in children and adolescents.

**Table:** 

**What is Known:** • *Current international pediatric blood pressure guidelines are based on percentile-based reference values derived from US multiethnic cohorts.* • *These reference values may not accurately reflect other populations, leading to potential misclassification.*
**What is New:** • *The novel Danish age- and sex-specific reference values, provided in this study, can be used in the assessment of oscillometric blood pressure measurements for screening purposes.* • *The novel oscillometric reference values are easy to apply and might reduce the risk of underdiagnosis.*

**Supplementary Information:**

The online version contains supplementary material available at 10.1007/s00431-026-06969-5.

## Introduction

Hypertension in children and adolescents is associated with long-term risk of cardiovascular disease; however, the condition is often underdiagnosed, and the prevalence is increasing [1–3]. A 2019 review showed an increase in childhood hypertension from 1.3% in the 1990 s to 6.0% between 2010 and 2014 globally [1]. Recent evidence further suggests that the risk profile of pediatric hypertension is evolving beyond traditional cardiometabolic determinants to encompass emerging behavioral and environmental exposures, including psychosocial stress, sleep deprivation, excessive screen use, and novel nicotine products, raising concern about a potential further increase in prevalence [4].

Assessment of blood pressure (BP) in children and adolescents requires interpretation of percentile-based age-, sex-, and height-specific reference values, as fixed outcome-based cut-offs for long-term risk are lacking within the pediatric population [5–7]. In children under 13 or 16 years old, with the age limit varying across guidelines (Table 1), hypertension is defined as auscultatory office BP ≥ 95th percentile for age, sex, and height in repeated measurements across clinical visits.

**Table 1:** Percentile-based reference values in the current international guidelines for hypertension in children and adolescents

Guideline	Reference	Dataset used for reference values	Cut-offs according to percentile	Fixed cut-off	
			Age group (year)	**Age group (year)**	**BP value (mmHg)**
AAP guideline	Flynn JT, 2017	Weight-modified US-based data	1–12	≥ 13	≥ 130/80
ESC guideline	De Simone G, 2022	Weight-modified US-based data	1–15	≥ 16	≥ 130/85
ESH guideline	Giuseppe M, 2023	Original US-based data (no weight criteria)	1–15	≥ 16	≥ 140/90

*AAP* American Academy of Pediatrics, *ESC* European Society of Cardiology, *ESH* European Society of Hypertension, *BP* blood pressure

All age groups are inclusive. Blood pressure was measured by the auscultatory method

Previous and current American [5] and European [6, 7] guidelines on hypertension in children and adolescents rely on percentile-based reference values based on auscultatory office BP measurements in a multiethnic US dataset, primarily collected between the 1970 s and 1980 s [8, 9]. However, due to differences and temporal changes in BP distributions across populations and over time, the US-based reference values may not be universally appropriate, as a recent southern and eastern European study has indicated [10].

The reference values in the European Society of Hypertension (ESH) guideline [6] are based on an original statistical model including children of all weights. Conversely, the 2017 American Academy of Pediatrics (AAP) guideline [5] and the 2022 European Society of Cardiology (ESC) guideline [7] are based on a modified statistical model including only normal-weight children [9] (Table 1).

Generally, standardized auscultatory office BP measurement is recommended for screening, diagnosis, and management of hypertension in children and adolescents [5, 11, 12]. Nevertheless, oscillometric office BP measurement is widely used in clinical practice due to its ease of use compared to the auscultatory method [13]. However, reference values based on oscillometric data are lacking [5–7, 11].

The primary aim of this study was to establish percentile-based population-specific oscillometric office BP reference values in normal-weight, ethnically Danish children and adolescents. Secondly, we evaluated the applicability of existing percentile-based international reference values in the assessment of oscillometric blood pressure measurements within this Danish pediatric cohort.

## Materials and methods

We conducted a cross-sectional study utilizing data from the Lolland–Falster Health Study (LOFUS) to generate percentile-based population-specific reference values for oscillometric office BP in children and adolescents [14]. To explore the potential for simplifying reference models, we examined the role of height in the modeling of BP across populations with varying heterogeneity; Data from the US National Health and Nutrition Examination Survey (NHANES) were used for this part [15]. To assess the applicability of existing international pediatric reference values, we used the novel Danish reference values as an internal comparative benchmark in reclassification analyses.

### LOFUS data

LOFUS is a Danish cohort study that randomly selected households in the rural area of Lolland–Falster, Denmark [14]. All individuals in these households were invited to participate. From 2016 to 2020, a total of 18,494 individuals aged 0–96 years were enrolled (2295 children aged 4–17) with a participation rate of 36% in general [14] and 28–32% in children above 1 year of age [16]. The data collection included age-specific questionnaires, physical examinations, and biological samples. LOFUS was approved by Region Zealand’s Ethical Committee on Health Research (SJ-421) and The Danish Data Protection Agency (REG-24–2015).

**Fig. 1:**
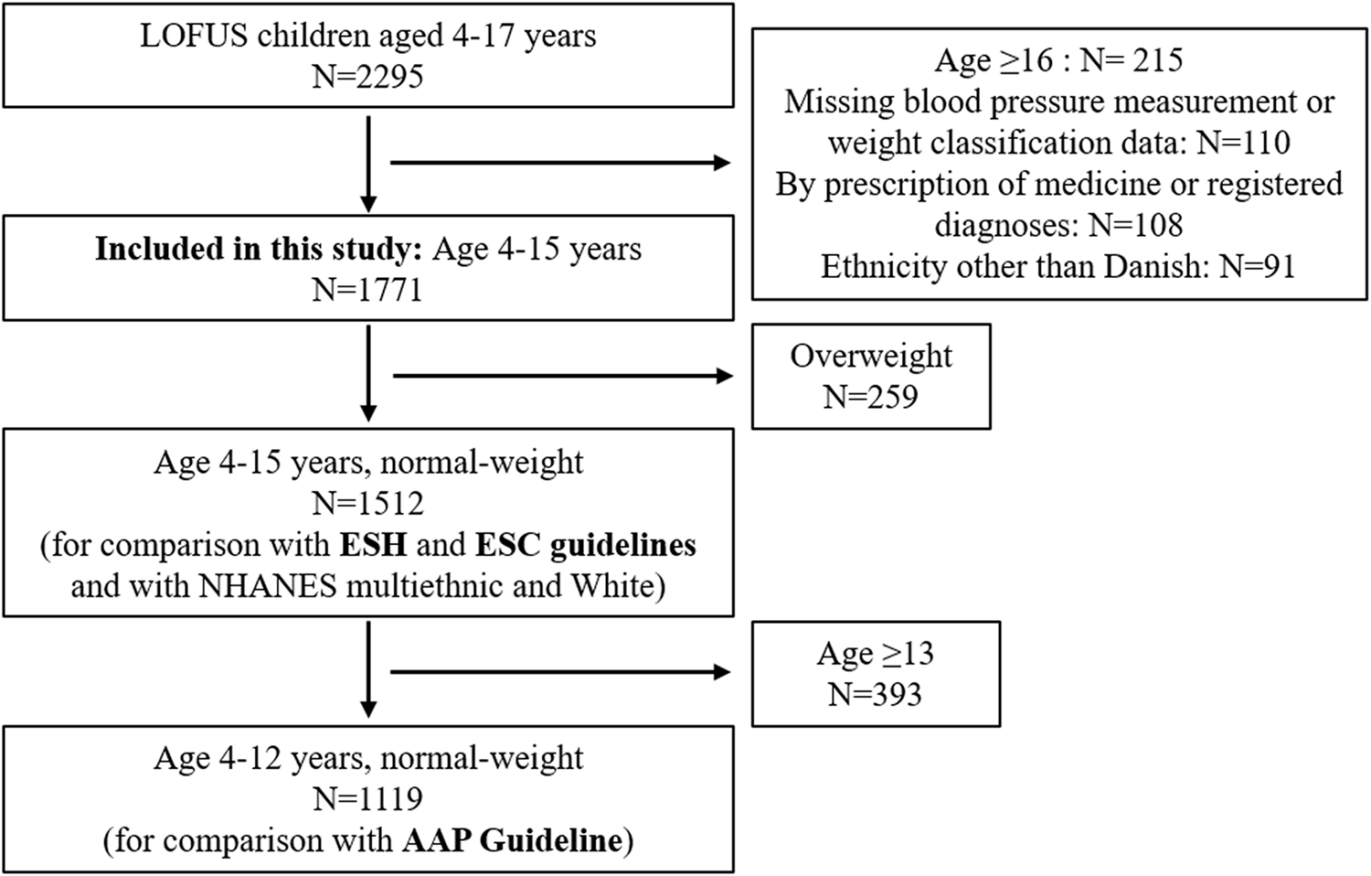
Flow chart for the inclusion of children from the Lolland–Falster Health Study (LOFUS)

The inclusion criterion for the present study was children aged 4–15 years from LOFUS (Fig. 1). Children were excluded if data on BP or for weight classification were not available or if they had redeemed prescriptions for relevant medicines or a relevant diagnosis recorded in the national registers (details in Supplementary Information). Furthermore, children were excluded if their country of origin was other than Denmark. This classification was based on register data on parental country of birth (for immigrants) and citizenship (for descendants). When information was available for both parents, the mother’s data were prioritized. If information was available for only one parent, that parent’s data were used. In the absence of parental information, the child’s own data were applied. Exclusions based on country of origin were performed to increase ethnic homogeneity within the study population. The small number of participants with a country of origin other than Denmark (48 boys and 43 girls, median age 9.4 (IQR, 6.5–12.5)) precluded meaningful analyses of blood pressure across other specific non-Danish groups. Based on this exclusion criterion, the included cohort was considered ethnically Danish.

### Measurements

BP was measured using the Welch Allyn Pro-BP-3400 electronic device (Welch Allyn, New York), which was validated against the auscultatory method for both children and adults, according to the British Society protocol, and met the AAMI-SP10 criteria [17]. BP was measured at a single visit in a quiet environment, with the participant in the supine position, arm supported, after a 5-min rest. The cuff size was selected based on measurement of the mid-upper arm circumference, as specified by the manufacturer (15–18 cm, child; 18–22 cm, size S; 22–32 cm, size M; 32–45 cm, size L). BP was measured three times, 1 min apart. Mean systolic and diastolic BP were used in the regression analyses and were defined as the average of the last two out of three measurements [5–7], which was available for 98% of the included children. All participants had at least one BP measurement recorded. If only one or two measurements were available, we used the average or the single measurement. Heart rate was measured using the Nellcor SpO2-D-YS (Mansfield, USA). For information on anthropometric measurements, see Supplementary Information.

### National Danish registers

Individual-level data from LOFUS were linked to national Danish health registers using each participant’s unique personal identification number (CPR-number), which is assigned to each citizen in Denmark at birth or immigration. Denmark has a tax-funded healthcare system that ensures equal access for all residents, and the CPR-system allows continuous coverage of hospital contacts (including registered ICD-10 diagnoses), redeemed prescriptions at pharmacies, and sociodemographic information. Data were obtained from the Danish National Prescription Registry [18], from the Danish National Patient Register [19], and from Statistics Denmark’s population registers.

### NHANES data

NHANES is a comprehensive population-based survey conducted by the Center for Disease Control and Prevention in the USA [15]. Data on health status is collected on a nationally representative sample and made publicly available. We included data on normal-weight children aged 4–15 years from the 2017–2018 data collection period, all of whom had BP data available [15]. The age range was selected to correspond to the LOFUS cohort, in order to maximize comparability between the two samples. We used data on sex, age, height, self-reported ethnicity (categorized as mixed ethnicity (all) or non-Hispanic White only) and oscillometric office BP (Omron IntelliSense BP Monitor). Cuff size selection was consistent with LOFUS. Up to three measurements were obtained after a 5-min rest [15]. Mean BP was calculated as described for LOFUS data. The differences between BP measurements in LOFUS and NHANES were as follows: (a) different devices used, and (b) supine position (LOFUS) versus the sitting position (NHANES).

### Statistics

All statistical analyses were conducted using R (Version 4.2.1, R-Core-Team, 2022) [20]. This study was reported following the STROBE guideline for cross-sectional studies [21].

### Descriptive statistics

Medians and interquartile ranges were used for descriptive statistics. Differences between sexes were tested using the Wilcoxon Rank-sum test. Body mass index (BMI) values were converted to standard deviation scores (SDS) based on growth charts from the International Obesity Task Force (IOTF) [22]. We used cut-offs for overweight corresponding to a BMI of 25 at age 18, as per the IOFT classifications of overweight. In case of missing data on weight (3.5%), overweight was defined as a waist-to-height ratio ≥ 0.5 [23].

### Analytical strategy

The adjusted coefficient of determination (*R*^2^) were compared across different statistical models of the association between systolic and diastolic BP and predictors (age, sex, and height): (a) linear regression, which models the BP as a linear combination of the predictors and (b) cubic spline, which allows for flexible modeling of a non-linear relationship (as done in previous studies [9]). Knots were placed at the 1 st quartile value, median, and 3rd quartile value of the independent variables. Based on the best fit of either (a) or (b), we proceeded with (c) a quantile regression, to be able to model how predictors directly affect specific quantiles of BP. Lastly, we compared our Danish office BP reference values with international reference values [5–7].

### Primary outcome

BP was modeled in univariate and multivariate linear regression analyses with and without interaction effects. Assumptions for linear regression were tested by QQ-plots and residual plots. The performance of the best linear regression model (as defined by the LOFUS data) was evaluated on the NHANES dataset.

Quantile regression models estimated BP percentiles, i.e., distribution-based reference values, at 5% intervals (5–95th percentile); these constituted the primary outcome in this study. The regression model was based on a linear relationship between the quantile of the BP and the predictors.

### Comparison of the novel Danish versus international reference values

We applied both the original American reference values [8] and the weight-modified version [9] to the LOFUS cohort. For each child, systolic and diastolic blood pressure percentiles were calculated separately, using the published regression equations underlying the respective models [8, 9].

We compared classifications derived from the international pediatric reference values with those based on the novel Danish reference values. The comparison was performed using reclassification tables, with the novel Danish model serving as an internal comparative benchmark. Screening principles, such as the Wilson–Jungner criteria, emphasize that a suitable screening test should minimize false negatives, to ensure cases are not missed [24]. However, acceptable thresholds vary across screening programs. In the present context, detection rates above 85% were considered acceptable.

In addition, we quantified differences between reference values across models by calculating the absolute difference (in mmHg) between the 95th percentile values for the novel Danish reference values and the international reference values for the given sex, age, and 50th height percentile.

## Results

This cross-sectional study provided percentile-based population-specific oscillometric office BP reference values based on children and adolescents of Danish origin. In addition, BP classification was compared to existing percentile-based international auscultatory reference values.

We included 1771 Danish children aged 4–15 years (887 girls and 884 boys) from LOFUS (Table 2). Only children of normal weight (*N* = 1512) were included in the regression models for defining reference values. Data on 655 children from NHANES were included to examine how blood pressure modeling is influenced by sample homogeneity (Table S1).

**Table 2:** Physical characteristics of included children from the Lolland–Falster Health Study (LOFUS), Denmark (total, *N*** = **1771)

	Age 4–12 years			Age 13–15 years		
	Girls	Boys	*p*-value	Girls	Boys	*p*-value
	*N* = 656	*N* = 640		*N* = 231	*N* = 244	
Age, years	9 (4)	9 (4)	0.7	15 (1)	15 (2)	0.4
Height, cm	136 (25)	138 (22)	0.4	166 (9)	173 (13)	< 0.001
Weight, kg	30 (16)	31 (15)	0.9	57 (12)	59 (16)	0.01
Waist, cm	60 (10)	61 (19)	0.005	71 (9)	74 (10)	< 0.001
Waist-to-height ratio	0.449 (0.07)	0.455 (0.06)	0.01	0.428 (0.06)	0.424 (0.05)	0.8
BMI, kg/m^2^	16 (4)	16 (3)	0.1	21 (5)	20 (3)	0.004
BMI SDS	0.14 (0.35)	0.008 (1.4)	0.1	0.42 (1.43)	0.25 (1.25)	0.31
Heart rate, min^−1^	83 (17)	80 (15)	< 0.001	72 (16)	70 (15)	0.007
Mean systolic BP, mmHg	103 (10)	103 (9)	0.4	110 (11)	112 (12)	0.001
Mean diastolic BP, mmHg	65 (6)	64 (6)	< 0.001	67 (7.5)	66 (8)	0.002

*BMI* body mass index (weight (kg)/height^2^(m)), *BMI SDS* BMI standard deviation score based on International Obesity Task Force (IOTF) growth charts, *BP* blood pressure

Values are medians and interquartile ranges. *p*-values are from Wilcox Rank-sum test

BP was measured in the supine position, arm supported, using Welch Allyn Pro-BP-3400 electronic device (Welch Allyn, New York). Mean BP was defined as the average of the last two out of three consecutive measurements

LOFUS represents a rural part of Denmark; however, the variability of height across age and sex strata in our sample was similar to that observed in the Danish growth reference population (*n* ≈ 7300) described by Tinggaard et al. [25], with standard deviations ranging from 3.6 to 8.5 cm, supporting the representativeness of the height distribution in our study sample.

### Assessing the association of age, sex, and height with BP

In univariate models, age and height were associated with both systolic and diastolic BP in LOFUS and NHANES data (Table 3 and Tables S2–S3). Age and height were highly correlated (*R*^2^, 0.90 (LOFUS), 0.75 (NHANES, mixed), and 0.77 (NHANES, non-Hispanic white). Female sex was associated with higher diastolic BP in LOFUS, but there was no significant association with systolic BP. Examination of interaction terms showed comparable adjusted *R*^2^ values, and the *R*^2^ of spline models were comparable to that of linear models (Figs. S2–3).

**Table 3:** Adjusted *R*^2^-values from the linear regression models of 1512 normal-weight Danish children aged 4–15 years

	Systolic blood pressure	Diastolic blood pressure
BP ~ age	0.263	0.062
BP ~ height	0.267	0.049
BP ~ sex	− 0.0004 (NS)	0.014
BP ~ age + height	0.272	0.063
BP ~ age + sex	0.262	0.076
BP ~ age + height + sex	0.271	0.076

*BP* blood pressure, *NS* not significant

BP was measured in the supine position, arm supported, using Welch Allyn Pro-BP-3400 electronic device (Welch Allyn, New York). Mean BP was defined as the average of the last two out of three consecutive measurements

Multivariate analysis showed that adding height to the model improved the variance explained in systolic and diastolic BP, in LOFUS, and non-Hispanic white NHANES data by 2.8–3.4% for systolic BP and 0.0–1.6% for diastolic BP (Table 3 and Table S3). In the mixed population NHANES data, the variance explained improved by 16.9% for systolic BP and 3.7% for diastolic BP.

In the LOFUS data, the full model estimated mean differences of 1.1 mmHg (maximum 1.8 mmHg) in the predicted 95th percentile of systolic BP between children at median height and those at the 5th or 95th height percentile, and 0.2 mmHg (maximum 0.3 mmHg) for diastolic BP. The simple model identified 98% of individuals with systolic and/or diastolic BP ≥ 95th percentile when compared with the full model, with only 0.3% reclassification, supporting omission of height in the model.

### The novel Danish reference values—primary outcome

The novel Danish reference values were based on a quantile regression model including age and sex, which enabled the estimation of reference values of oscillometric office BP from the 5th to 95th percentile (Table 4 and Tables S4–S5). Analyses revealed that the spread of systolic BP values changed significantly with age in both girls and boys (i.e., the variance was not constant over time), such that older children tended to show a wider range of systolic BP values, indicating more biological heterogeneity or age-dependent measurement issues. In contrast, the spread of diastolic BP values stayed more constant.

**Table 4:** Age and sex-specific 50th, 90th, and 95thpercentile reference values of oscillometric office blood pressure (mmHg) in 1512 Danish children aged 4–15 years

	Girls						Boys					
	Systolic blood pressure			Diastolic blood pressure			Systolic blood pressure			Diastolic blood pressure		
Age (years)	50th	90th	95th	50th	90th	95th	50th	90th	95th	50th	90th	95th
4	96	104	107	62	69	70	96	104	109	61	67	69
5	97	105	108	63	69	71	97	106	110	62	68	69
6	99	107	110	63	70	71	99	107	111	62	68	70
7	100	109	112	64	70	72	100	109	113	63	69	70
8	101	110	113	64	71	72	101	111	114	63	69	71
9	103	112	115	65	71	73	103	112	116	64	70	71
10	104	114	116	65	72	73	104	114	118	64	70	72
11	105	115	118	66	73	74	105	116	119	65	71	72
12	107	117	120	66	73	74	107	117	121	65	71	73
13	109	119	121	66	73	75	108	119	122	65	71	73
14	110	120	123	67	74	75	109	120	124	66	72	74
15	111	122	124	67	74	76	111	122	126	66	72	74

Blood pressure was measured in the supine position, arm supported, using Welch Allyn Pro-BP-3400 electronic device (Welch Allyn, New York). Mean blood pressure was defined as the average of the last two out of three consecutive measurements

Danish ethnicity was defined by country of origin

### Comparison with international reference values

Using the novel Danish reference values as an internal comparative benchmark, the reclassification analyses showed that only 69%, 56%, and 27% of children with office BP ≥ 95% according to the Danish reference were similarly classified when applying international reference values according to the AAP, ESC, and ESH guidelines, respectively (Table 5). False negative and false positive rates were 2–6% and 0–1%, respectively. While the reclassification of girls was lower compared to boys when applying the AAP guideline, the reverse pattern was observed when applying the ESC and ESH guidelines.

**Table 5:** Reclassification tables comparing the classification according to systolic and/or diastolic blood pressure ≥ 95th percentile when using the novel Danish reference values versus the international reference values

	Guideline:	American Academy of Pediatrics (Flynn, 2017)		European Society of Cardiology (De Simone G, 2022)		European Society of Hypertension, (Working group, 2023)	
	Age	< 13 years (*N* = 1119)		< 16 years (*N* = 1512)		< 16 years (*N* = 1512)	
	Percentile	≥ 95th	< 95th	≥ 95th	< 95th	≥ 95th	< 95th
The novel Danish reference values (Mikkelsen LF, 2025)	≥ 95th	57	26	66	51	32	85
	< 95th	9	1027	9	1386	0	1395
Comparison of blood pressure classification at the ≥ 95th percentile*	All	57/(57 + 26) = 69%		66/(66 + 51) = 56%		32/(32 + 85) = 27%	
	Girls	64%		63%		36%	
	Boys	74%		49%		19%	

Percentile ≥ 95th: Systolic *and/or* diastolic blood pressure percentile equal to or above the 95th percentile

Percentile < 95th: Systolic *and* diastolic blood pressure percentile below the 95th percentile

^*^The novel Danish reference values were used as an internal reference in this analysis

International reference values were based the auscultatory measurements of office blood pressure in the upright sitting position

The novel Danish reference values were based on oscillometric blood pressure measurements in the supine position, arm supported, using Welch Allyn Pro-BP-3400 electronic device (Welch Allyn, New York)

Furthermore, 77%, 64%, and 38% of children with office BP ≥ 90% according to the novel Danish reference values were similarly classified when applying the AAP, ESC, and ESH guidelines, respectively (Table S6). In overweight children, 50%, 65%, and 40% of children with office BP ≥ 95th percentile according to the Danish reference were similarly classified when applying the AAP, ESC, and ESH guideline, respectively (Table S7).

Reference values reported in this study were generally lower than the US-based international reference values. Differences varied from + 1 to − 9 mmHg, were more pronounced in diastolic as compared to systolic BP, and increased with age (Table S8). The disagreement is illustrated with Bland–Altman plots in Fig. S3.

## Discussion

This study provided percentile-based population-specific reference values for oscillometric office blood pressure screening in Danish children. Application of international reference values, with the novel Danish model used as an internal comparative benchmark, resulted in reclassification of blood pressure status. Agreement in classification was limited across both normal-weight and overweight children. These findings have potential implications for blood pressure screening in children and adolescents by providing simplified oscillometric reference values and suggesting that caution is warranted when applying international blood pressure reference values across different pediatric populations and measurement modalities.

Analyses showed that, in a homogeneous pediatric population, office BP reference values can be modeled using age and sex alone, without compromising the explanatory value of the model when omitting height.

The higher rate of false negatives than false positives indicates that the international reference values classify fewer individuals as having blood pressure ≥ 95th percentile than the novel Danish model. This may imply that children who may warrant further evaluation could be overlooked when international reference values are applied in a Danish context, when oscillometric devices are used for screening. Some of the observed discordance between the Danish and the international reference values may be due to differing measurement methods, auscultatory versus oscillometric. However, based on data from eight Southern and Eastern European countries, Lurbe et al. [10] found that pediatric oscillometric office BP values were generally *higher* than auscultatory values. And when compared to the American values [8, 9] used in current guidelines, the multiethnic European reference values were also generally higher. In our study, we were unable to validate our results against auscultatory measurements; however, the findings by Lurbe et al. suggest that the difference we observed, where oscillometric readings in the Danish LOFUS study are *lower* than the American auscultatory values, is unlikely to be attributed to measurement method alone. A Swedish [26] study using oscillometric office BP measurements reported normative values comparable to those found in our study, with 95th percentile ranges of 115–124/71–76 mmHg (systolic/diastolic) in girls and 115–131/70–75 mmHg in boys across the age span of 6–15 years. The strong agreement with reference values from another Scandinavian population further supports the validity of our findings.

We speculate that the lower office BP percentiles in the Danish cohort may partly reflect the following: (1) inherent and secular differences between populations, including temporal differences between data collection periods spanning several decades, as well as variation in genetic factors [27], socioeconomic conditions [28], dietary patterns, physical activity levels, and other cardiometabolic risk profiles, including differences in the prevalence of obesity across countries; and (2) our ability, by using national health registers, to apply stringent exclusion criteria and thereby include only healthy children with expectedly normal BP. Similar exclusion procedures were not evident in the international cohorts. Moreover, as mentioned above, differences between the auscultatory and oscillometric methods cannot be ruled out, and using the supine position in contrast to the sitting upright position during office BP measurement may also have affected our results [29].

### Multiethnic versus ethnically homogeneous population

Height is a heritable trait that varies widely across continents and within European countries [27]. Neither Lurbe et al. [10] nor any of the statistical models that form the basis for the current guidelines [8, 9] have taken ethnicity-related confounding into account in their equations. All previous models found that adding height improved models more than we can explain in this Danish study, where adding height to the models for systolic and diastolic BP improved the explanatory value by only 0–3.4%. Adding height to the model in non-Hispanic white NHANES improved the explanatory value by 0.4–2%, and in mixed NHANES the model improved by 16.9% for systolic BP and 3.7% for diastolic BP. Hence, the explanatory value of height was markedly lower in the more ethnically homogeneous population. This might be explained by heterogeneity in growth patterns, nutritional status, and pubertal timing and supports our findings in the Danish cohort.

Our results highlight the importance of accounting for population heterogeneity, including ethnicity, when developing reference values for BP. This may be attempted by either conducting analyses in ethnically homogeneous populations, as in this paper, or by explicitly including ethnicity as a variable in the modeling process. The latter approach would enable the development of international multi-ethnic reference values, but this would require substantially larger sample sizes and the introduction of additional covariates might complicate clinical assessment. Selection of covariates—such as height, age, sex, ethnicity and potentially weight—should be guided by their relevance and predictive value within the specific dataset. Notably, the relevance of height-adjusted models may differ in settings with broader anthropometric variability, and the impact of sex on blood pressure in our models may have been reduced by stringent exclusion criteria, as sex-related differences could be attenuated through this process.

### Strengths and limitations

We included a large, population-based cohort with oscillometric office BP measurements at a single visit in children across a wide age range, but were unable to determine the true prevalence of sustained BP elevation or intra-individual variation. Due to the cross-sectional design, we were unableto validate oscillometric office BP against auscultatory office BP or ABPM, nor to evaluate our findings in relation to long-term clinical outcomes. ABPM is recommended for confirmation of diagnosis, and assessment could have added valuable insight into BP patterns and white-coat effect. The aim was to establish percentile-based Danish pediatric oscillometric office BP reference values, and results cannot be generalized to other populations (country of origin other than Denmark), or measurement methods (oscillometric measurement, in supine position using Welch Allyn Pro-BP-3400 electronic device, Welch Allyn, New York). Hence, while the exclusion of children with a country of origin other than Denmark increased the homogeneity of the sample, it limited the external generalizability. Gender identity was not assessed in this study, and it is noteworthy that the applicability of our reference values to children and adolescents whose gender identity differs from their sex assigned at birth may be limited. Furthermore, the reference values in this study are based on a single-visit oscillometric blood pressure measurement and should therefore be interpreted as a screening distribution rather than a diagnostic threshold, as diagnosis of hypertension would require confirmatory auscultatory measurements and/or ABPM.

We were unable to assess the applicability of the international guidelines or the novel Danish models against an established benchmark in a Danish population, as no such standard exists. Therefore, the novel Danish model was used as an internal comparative benchmark in the reclassification analyses.

### Clinical implications

There is a pressing need for reference values, as presented in this study, for the assessment of pediatric oscillometric office blood pressure in screening, as oscillometric BP measurements are now standard practice for BP screening in many clinical settings [13]. The novel Danish percentile-based pediatric reference values, based solely on age and sex, provide a simplified and more user-friendly approach to office BP assessment in children, relying on fewer covariates. This approach is further facilitated by the ease of oscillometric measurements compared with the auscultatory method, which requires more operator experience. Importantly, this simplification carries only minimal risk of misclassification (0.3%), including children at all height percentiles. Rea C. J. et al. [13] showed that BP screening in children is often not carried out in accordance with current guidelines, partly due to the complexity of the diagnostic tools currently recommended, underscoring the relevance of a more easy-to-use assessment tool. However, as noted above, the external generalizability of the reference values presented in this study is limited to Danish children (defined as having Denmark as the country of origin) and to blood pressure measurements performed according to the procedures and specific device used in this study.

In Denmark, the potential risk of misclassification of office BP due to the use of non-applicable international reference values, when using oscillometric devices for screening purposes, may contribute to the persistently high level of underdiagnosed pediatric hypertension [30]. At the international level, the applicability of multiethnic reference values may also be limited in populations beyond Denmark, there is a clear need to further investigate this risk. Early and accurate detection of elevated BP is critical, as childhood hypertension is a modifiable risk factor for adverse cardiovascular trajectories in adulthood [31–35]. Moreover, global challenges in implementing recommendations for routine office BP screening in children [11] underscore the relevance of our findings. The use of simplified, population-specific reference values may support more effective clinical practice across both primary and secondary healthcare settings.

Future research should focus on validating our model, in larger national cohorts and examining its predictive value for long-term cardiovascular outcomes, as no outcome-based cut-off values have yet been established for pediatric populations.

In conclusion, this study provides novel percentile-based reference values for oscillometric office blood pressure screening in in children and adolescents of Danish origin. Relying on international reference values to assess oscillometric office BP in Danish children may carry risk of misclassification and, consequently, underdiagnosis—a concern that may also apply in other populations.

## Supplementary Information

Below is the link to the electronic supplementary material.

ESM 1 (PDF 1.41 MB)

## Data Availability

The LOFUS data that support the findings of this study are not publicly available due to data protection regulations but may be made available upon reasonable request and with permission from the LOFUS steering committee. The NHANES data used in this study are publicly available from the National Health and Nutrition Examination Survey (NHANES) website.

## Abbreviations

AAP: American Academy of Pediatrics
ABPM: 24-H ambulatory blood pressure monitoring
BP: Blood pressure
BMI: Body mass index
CPR: Civil personal registration
ESC: European Society of Cardiology
ESH: European Society of Hypertension
ICD-10: International Classification of Diseases, 10th edition
IOTF: International Obesity Task Force
LOFUS: Lolland–Falster Health Study
NHANES: National Health and Nutrition Examination Survey
SDS: Standard deviation score
