# Supplementary material for: A novel model for percentile-based oscillometric blood pressure reference values in Danish children: implications for the use of international standards in pediatric screening
Source: Eur J Pediatr. 2026 Apr 30;185(5):326. doi: 10.1007/s00431-026-06969-5 (PMC13132949; doi:10.1007/s00431-026-06969-5)
Supplement: Supplementary file 1 — (PDF 1.41 MB) [file 431_2026_6969_MOESM1_ESM.pdf]

Supplementary Information

Article title: A novel model for percentile-based oscillometric blood pressure reference values in Danish children: implications for the use of international standards in pediatric screening

Journal name: European Journal of Pediatrics

Author names: Lise Fischer Mikkelsen, MD; Henrik Enghusen Poulsen, MDsc; Mikkel Porsborg Andersen, PhD; Søren Hagstrøm, PhD; Konstantinos Kamperis, PhD; *Jørgen Kim Kanters, PhD; *Christina Ellervik, MDsci; *Luise Borch, PhD *shared last authors

Corresponding Author: Lise Fischer Mikkelsen
Department of Clinical Medicine, Aarhus University, Aarhus, Denmark;
Department of Pediatric and Adolescent Medicine, Gødstrup Hospital, 7400 Herning, Denmark.
Mail: lisehr@rm.dk

Table of Content

Exclusion criteria and anthropometric measurements 2

Figure S1 Distributions of oscillometric systolic and diastolic blood pressure (N=1771) 2

Table S1: Basic characteristics in normal-weight LOFUS children aged 4-15 years and normal-weight NHANES (2017-2018) children aged 4-15 years, mixed ethnicity and white 3

Table S2: Variance explained (adjusted R^2 values) of oscillometric blood pressure measurements in linear models in normal weight children aged 4-15 years from the Danish LOFUS study and the American NHANES study (2017-2018) 4

Table S3: Comparison of linear regression models in normal-weight Danish children aged 4-15 years from the LOFUS study and normal-weight American children aged 4-15 from NHANES 2017-2018 5

Figure S2: Linear regression models (left) and spline models (right) for blood pressure as a function of age in Danish girls in the LOFUS study. 6

Figure S3: Linear regression models (left) and spline models (right) for blood pressure as a function of age in Danish boys in the LOFUS study. 6

Table S4: Regression coefficients from quantile regression models of systolic and diastolic blood pressure as a function of age and sex in 1512 Danish Children from the LOFUS study. 7

Table S5A: Percentile values of oscillometric office blood pressure (mmHg) in girls aged 4-15 years 8

Table S5B: Percentile values of oscillometric office blood pressure (mmHg) in boys aged 4-15 years 9

Table S6. Reclassification tables comparing the novel Danish reference model (oscillometric) versus the diagnostic decision limits (blood pressure ≥90th vs. <90) in international guidelines (ausculatatory) 10

Table S7. Reclassification tables comparing the novel Danish model (oscillometric) versus the diagnostic criteria in international guidelines (auscultatory) in overweight children 11

Table S8: Difference in blood pressure (mmHg) reference value at the 95th percentile between the novel Danish model (oscillometric) and international guidelines (auscultatory) 12

Exclusion criteria and anthropometric measurements

Exclusion criteria: Children were excluded if data on blood pressure or data for weight classification (height and weight or waist circumference) were not available. Furthermore, individuals were excluded (A)if they had redeemed any of the following prescriptions: drugs used in diabetes (ATC: A10), cardiovascular system including antihypertensives (ATC: C01 – C10), thyroid therapy (ATC: H03), antimigraine medication (ATC: N02C), or psychoanaleptics (ATC: N06) and (B)if they had a documented hospital contact related to any of the following ICD-10 diagnosis within three years prior to LOFUS (Lolland Falster Health Study) enrolment: DC00–DC99 (malignant neoplasms), DD50–DD89 (diseases of the blood, blood-forming organs, and certain disorders involving the immune system), DE00–DE90 (endocrine, nutritional, and metabolic diseases), DI00–DI99 (diseases of the circulatory system), DN00–DN89 (renal diseases), or Q20–Q28 and Q60–Q64 (congenital malformations of the circulatory and urinary systems. Furthermore, children were excluded if they had a country of origin other than Denmark (see the main manuscript for definition).

Anthropometrics: Height was measured in centimeters (cm) without shoes in the standing position using the SECA 216 wall-mounted stadiometer (SECA, Chino, USA). Weight was measured in kilograms (kg) using the Tanita WB.110A digital medical scale. Most participants wore light clothes during weighing. For children aged 4-6 years, 0.5kg was subtracted before entry into the database, unless they were weighed naked. For children ≥7 years, 1 kg was subtracted. Waist circumference was measured to the nearest centimeter (cm) mid-point between the subcostal and suprailiac landmarks at end-expiration, in the standing position, using an inelastic measuring tape. BMI was calculated as weight/height^2 (kg/m^2).

Figure S1 Distributions of oscillometric systolic and diastolic blood pressure (N=1771)

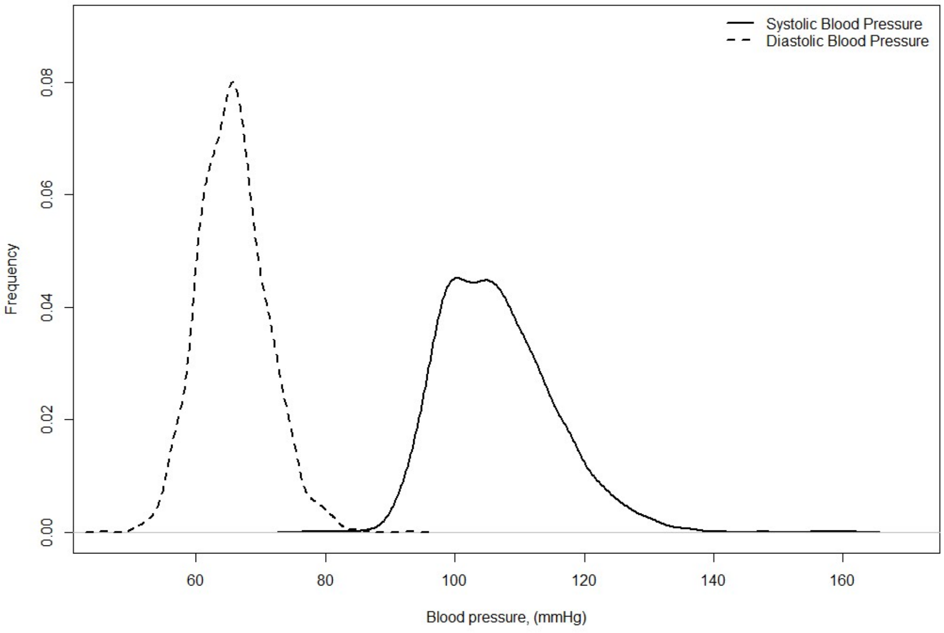

BP was measured in the supine position, arm supported, using Welch Allyn Pro-BP-3400 electronic device (Welch Allyn, New York). Mean BP was defined as the average of the last two out of three consecutive measurements.

Table S1: Basic characteristics in normal-weight LOFUS children aged 4-15 years and normal-weight NHANES (2017-2018) children aged 4-15 years, mixed ethnicity and white

	LOFUS	NHANES mixed	NHANES white
N	1512	655	191
Girls, N(%)	748(49)	306(47)	84(47)
Age, years	10.5(5.3)	11(4)	11(4)
Height, cm	144(33)	147(24)	147(22)
Weight, kg	32(23)	38(19)	37(20)
Waist, cm	62(12)	64(11)	64(10)
BMI, kg/cm^2	17(4)	17(3)	17(3)
Systolic Blood Pressure	104(12)	102(11)	101(10)
Diastolic Blood Pressure	65(7)	56(11)	55(11)

Values are medians and interquartile ranges.
LOFUS: Lolland-Falster Health Survey
NHANES: National Health and Nutrition Examination Survey

BP was oscillometric office measurements. The differences between BP measurements in LOFUS and NHANES were: (a) different devices used, and (b) supine position (LOFUS) versus the sitting position (NHANES).

Table S2: Variance explained (adjusted R^2 values) of oscillometric blood pressure measurements in linear models in normal weight children aged 4-15 years from the Danish LOFUS study and the American NHANES study (2017-2018)

	Systolic Blood Pressure (mmHg)			Diastolic Blood Pressure (mmHg)		
	LOFUS<br><br>N=1512	NHANES<br>(mixed)<br>N=655	NHANES<br>(white)<br>N=191	LOFUS<br><br>N=1512	NHANES<br>(mixed)<br>N=655	NHANES<br>(white)<br>N=191
BP ~ Age	0.263	0.118	0.145	0.062	0.159	0.246
BP ~ Height	0.267	0.138	0.142	0.049	0.152	0.215
BP ~ Sex	-0.0004 (NS)	0.008(NS)	-0.005(NS)	0.014	0.002(NS)	0.002(NS)
BP ~ Age + Height	0.272	0.138	0.149	0.063	0.165	0.246
BP ~ Age + Sex	0.262	0.125	0.141	0.076	0.162	0.244
BP ~ Age + Height + Sex	0.271	0.142	0.145	0.076	0.171	0.245

LOFUS: Lolland-Falster Health Survey
NHANES: National Health and Nutrition Examination Survey

NS: Not significant

BP was oscillometric office measurements. The differences between BP measurements in LOFUS and NHANES were: (a) different devices used, and (b) supine position (LOFUS) versus the sitting position (NHANES).

Table S3: Comparison of linear regression models in normal-weight Danish children aged 4-15 years from the LOFUS study and normal-weight American children aged 4-15 from NHANES 2017-2018

	LOFUS			NHANES (ALL)			NHANES (Non-Hispanic White)		
	N=1512			N=655			N=191		
	coefficient	SE	p-value	coefficient	SE	p-value	coefficient	SE	p-value
SBP									
Intercept	81.6	2.5	<0.001	76.6	4.0	<0.001	77.0	7.4	<0.001
Age(years)	0.55	0.17	0.395	0.27	0.3	0.2	0.8	0.5	0.1
Height(cm)	0.12	0.02	<0.001	0.15	0.04	<0.001	0.10	0.08	0.2
Sex(male=1, female=2)	0.09	0.37	0.82	-1.22	0.6	0.049	-0.18	-0.15	0.9
R^2-value	0.271			0.142			0.145		
DBP									
Intercept	60.6	1.75	<0.001	-11.5	8.9	0.2	-9.4	14.8	0.5
Age(years)	0.49	0.12	<0.001	1.9	0.6	0.002	2.9	1.0	0.006
Height(cm)	0.02	0.02	0.38	0.25	0.09	0.006	2.2	2.3	0.435
Sex(male=1, female=2)	1.20	0.25	<0.001	3.08	1.4	0.02	0.18	0.2	0.3
R^2-value	0.076			0.171			0.245		

Multivariate models include age, height, and sex (1: male, 2: female)
LOFUS: Lolland-Falster Health Survey
NHANES: National Health and Nutrition Examination Survey

SBP: systolic blood pressure

DBP: diastolic blood pressure

BP was oscillometric office measurements. The differences between BP measurements in LOFUS and NHANES were: (a) different devices used, and (b) supine position (LOFUS) versus the sitting position (NHANES).

Figure S2: Linear regression models (left) and spline models (right) for blood pressure as a function of age in Danish girls in the LOFUS study.

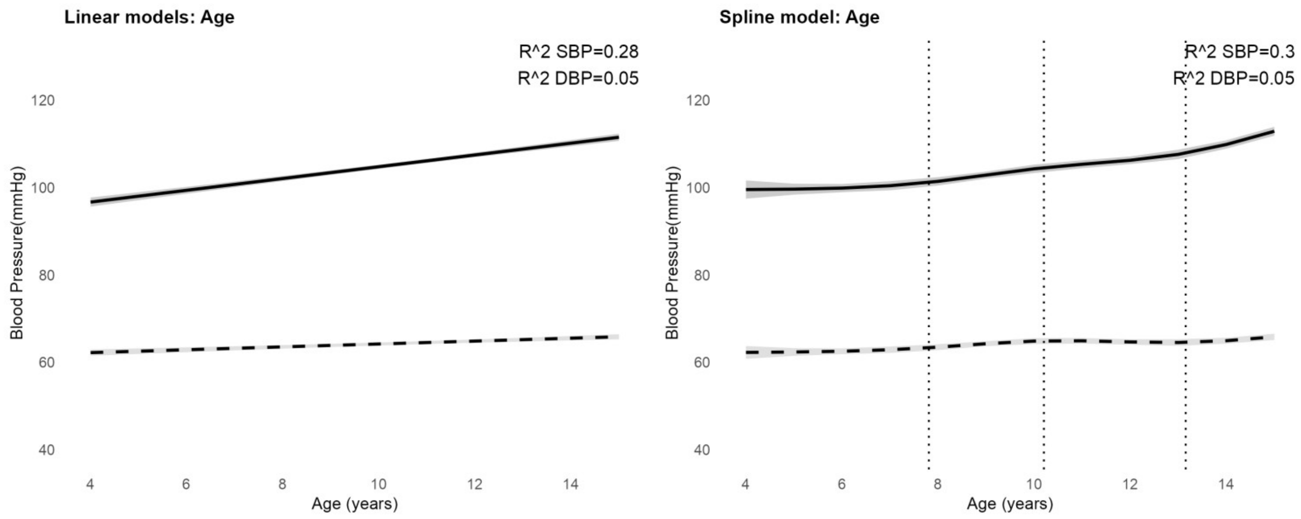

LOFUS: Lolland-Falster Health Survey. Solid line: systolic blood pressure. Dashed line: diastolic blood pressure.

Figure S3: Linear regression models (left) and spline models (right) for blood pressure as a function of age in Danish boys in the LOFUS study.

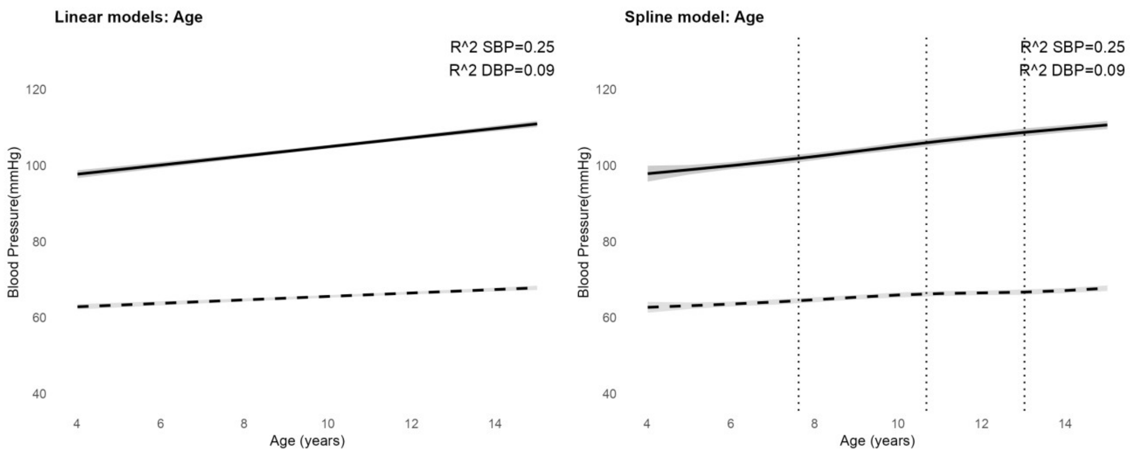

LOFUS: Lolland-Falster Health Survey. Solid line: systolic blood pressure. Dashed line: diastolic blood pressure.
SBP: Systolic blood pressure
DBP: Diastolic blood pressure
R^2: Adjusted R-squared
BP was measured in the supine position, arm supported, using Welch Allyn Pro-BP-3400 electronic device (Welch Allyn, New York).
Mean BP was defined as the average of the last two out of three consecutive measurements.

Table S4: Regression coefficients from quantile regression models of systolic and diastolic blood pressure as a function of age and sex in 1512 Danish Children from the LOFUS study.

		Systolic Blood Pressure			Diastolic Blood Pressure		
		τ=0.50	τ=0.90	τ =0.95	τ =0.50	τ=0.90	τ =0.95
β_0	Intercept	90.3	97.8	102.6	58.8	63.8	65.6
β_1	Age (years)	1.3	1.6	1.5	0.44	0.47	0.50
B_2	Sex (male=1, female=2)	0.22	-0.29	-1.12	0.93	1.6	1.40

τ =tau (quantile level)
LOFUS: Lolland-Falster Health Survey
BP was measured in the supine position, arm supported, using Welch Allyn Pro-BP-3400 electronic device (Welch Allyn, New York). Mean BP was defined as the average of the last two out of three consecutive measurements.

Table S5A: Percentile values of oscillometric office blood pressure (mmHg) in girls aged 4-15 years

Age:	Percentile:	5	10	15	20	25	30	35	40	45	50	55	60	65	70	75	80	85	90	95
4	SBP pct.	91	92	93	93	94	95	95	95	96	96	97	97	98	99	100	101	102	104	107
	DBP pct.	56	58	59	59	60	61	61	62	62	62	63	64	64	65	66	66	67	69	70
5	SBP pct.	91	93	94	94	95	96	96	96	97	97	98	99	99	100	102	102	104	105	108
	DBP pct.	57	59	59	60	61	61	62	62	62	63	63	64	65	65	66	67	68	69	71
6	SBP pct.	92	93	94	95	96	97	97	98	98	99	99	100	101	102	103	104	106	107	110
	DBP pct.	57	59	59	60	61	62	62	62	63	63	64	64	65	66	67	67	68	70	71
7	SBP pct.	93	94	95	96	97	98	98	99	100	100	101	101	102	103	105	105	107	109	112
	DBP pct.	57	59	60	61	61	62	62	63	63	64	64	65	66	67	67	68	68	70	72
8	SBP pct.	94	95	96	97	98	99	99	100	101	101	102	103	104	105	106	107	109	110	113
	DBP pct.	58	59	60	61	61	62	63	63	64	64	65	65	66	67	67	68	69	71	72
9	SBP pct.	95	96	97	98	99	100	100	101	102	103	104	104	105	106	108	108	110	112	115
	DBP pct.	58	60	60	61	62	62	63	64	64	65	65	66	66	67	68	69	70	71	73
10	SBP pct.	95	97	98	99	100	101	101	103	103	105	105	106	107	108	109	110	112	114	116
	DBP pct.	58	60	61	61	62	63	63	64	65	65	66	66	67	68	68	69	70	72	73
11	SBP pct.	96	98	99	100	101	102	102	104	105	106	106	107	108	109	111	112	114	115	118
	DBP pct.	58	60	61	61	63	63	64	64	65	66	66	67	67	68	69	70	71	72	74
12	SBP pct.	97	98	100	100	102	103	103	105	106	107	108	109	110	110	112	113	115	117	120
	DBP pct.	59	60	61	61	63	64	64	65	65	66	66	67	68	68	69	70	71	73	74
13	SBP pct.	98	99	100	101	103	104	104	106	107	108	109	110	111	112	114	115	117	119	120
	DBP pct.	59	60	62	63	63	64	65	65	66	66	67	68	68	69	70	71	72	73	75
14	SBP pct.	99	100	101	102	104	105	105	107	108	110	110	111	113	113	115	116	118	120	123
	DBP pct.	59	61	62	63	64	64	65	66	66	67	67	68	69	69	70	71	72	74	75
15	SBP pct.	99	101	102	103	105	106	107	109	110	111	112	113	114	115	117	118	120	122	125
	DBP pct.	59	61	62	63	64	65	65	66	67	67	68	69	69	70	70	71	73	74	76

SBP: Systolic Blood Pressure
DBP: Diastolic Blood Pressure

BP was measured in the supine position, arm supported, using Welch Allyn Pro-BP-3400 electronic device (Welch Allyn, New York). Mean BP was defined as the average of the last two out of three consecutive measurements.

Table S5B: Percentile values of oscillometric office blood pressure (mmHg) in boys aged 4-15 years

Age:	Percentile:	5	10	15	20	25	30	35	40	45	50	55	60	65	70	75	80	85	90	95
4	SBP pct.	90	92	93	94	94	95	95	95	96	96	96	97	98	99	100	101	102	104	108
	DBP pct.	55	56	58	58	59	60	60	61	61	61	62	63	63	64	65	65	66	67	69
5	SBP pct.	91	93	94	95	95	96	96	96	97	97	98	99	99	100	102	103	104	106	110
	DBP pct.	55	57	58	59	59	60	61	61	61	62	63	63	64	64	65	66	66	68	69
6	SBP pct.	92	94	95	96	96	97	97	97	98	99	99	100	101	102	103	104	106	107	111
	DBP pct.	55	57	58	59	60	61	61	61	62	62	63	64	64	65	66	66	67	68	70
7	SBP pct.	93	94	95	96	97	98	87	99	99	100	101	102	102	103	105	106	107	109	113
	DBP pct.	55	57	58	60	60	61	61	62	62	63	63	64	65	65	66	67	67	69	71
8	SBP pct.	93	95	96	97	98	99	99	100	101	101	102	103	104	105	106	107	109	111	114
	DBP pct.	56	57	59	60	60	61	62	62	63	63	64	64	65	66	66	67	68	69	71
9	SBP pct.	94	96	97	98	99	100	101	101	102	103	103	104	105	106	108	109	110	112	116
	DBP pct.	56	58	59	60	61	62	62	63	63	64	64	65	66	66	67	68	68	70	71
10	SBP pct.	95	97	98	99	100	101	102	102	103	104	105	106	107	108	109	110	112	114	118
	DBP pct.	56	58	59	61	61	62	62	63	64	64	65	65	66	67	67	68	69	70	72
11	SBP pct.	96	98	99	100	101	102	103	104	104	105	106	107	108	109	111	112	114	116	119
	DBP pct.	57	58	60	61	62	62	62	63	64	65	65	66	66	67	68	68	69	71	72
12	SBP pct.	97	98	100	101	102	103	104	105	106	107	107	109	110	111	112	113	115	117	121
	DBP pct.	57	58	60	61	62	62	63	64	64	65	66	66	67	67	68	69	70	71	73
13	SBP pct.	97	100	101	102	103	104	105	106	107	108	109	110	111	112	114	115	117	119	122
	DBP pct.	57	59	61	61	62	63	64	64	65	6665	66	67	67	68	68	69	70	71	73
14	SBP pct.	98	100	101	103	104	105	106	107	108	109	110	112	113	114	115	116	119	120	124
	DBP pct.	57	59	61	62	62	63	64	65	65	66	67	68	68	68	69	70	71	72	74
15	SBP pct.	99	101	102	104	105	106	108	109	109	111	112	113	114	115	117	118	120	122	126
	DBP pct.	58	59	61	62	63	63	64	65	66	66	67	68	69	69	69	70	71	72	74

SBP: Systolic Blood Pressure
DBP: Diastolic Blood Pressure

BP was measured in the supine position, arm supported, using Welch Allyn Pro-BP-3400 electronic device (Welch Allyn, New York). Mean BP was defined as the average of the last two out of three consecutive measurements.

Table S6. Reclassification tables comparing the classification according to systolic and/or diastolic blood pressure ≥90th percentile when using the novel Danish reference values (oscillometric) versus the international reference values (ausculatatory)

	Guideline:	**American Academy of Pediatrics** (Flynn, 2017)		**European Society of Cardiology** (De Simone G, 2022)		**European Society of Hypertension,** (Working group, 2023)	
	Age:	<13 years (N=1119)		<16 years (N=1512)		<16 years (N=1512)	
	Percentile:	≥90th	<90th	≥90th	<90th	≥90th	<90th
**The novel Danish model** (Mikkelsen LF, 2025)	≥90th	121	37	143	79	85	137
	<90th	36	925	39	1251	0	1290
**Comparison***	All	121/(121+37)=77%		143/(143+79)=64%		85/(85+137)=38%	
	Girls	85%		79%		50%	
	Boys	68%		53%		28%	

Percentile <90th: Systolic *and* diastolic blood pressure percentile below the 90th percentile.
Percentile ≥90th: Systolic *and/or* diastolic blood pressure percentile equal to or above the 90th percentile.
*The novel Danish reference values were used as an internal reference in this analysis.
International reference values were based the auscultatory measurements of office blood pressure in the upright sitting position.
The novel Danish reference values were based on blood pressure measurements in the supine position, arm supported, using Welch Allyn Pro-BP-3400 electronic device (Welch Allyn, New York).

Table S7. Reclassification tables comparing the classification according to systolic and/or diastolic blood pressure ≥95th percentile when using the novel Danish reference values (oscillometric) versus the international reference values (ausculatatory) in overweight children

	Guideline:	**American Academy of Pediatrics** Flynn, 2017		**European Society of Cardiology** De Simone G, 2022		**European Society of Hypertension,** Working group, 2023	
	Age:	<13 years<br>N=177		<16 years<br>N=259		<16 years<br>N=259	
	Percentile:	≥95th	<95th	≥95th	<95th	≥95th	<95th
**The novel Danish model** (Mikkelsen LF, 2025)	≥95th	8	8	14	8	9	13
	<95th	≤5	≥156	≤5	≥232	≤5	≥232
Comparison*	All:	8/(8+8)=50%		14/(14+8)=64%		9/(9+13)=40%	

Percentile ≥95th: Systolic *and/or* diastolic blood pressure percentile equal to or above the 95th percentile.
Percentile <95th: Systolic *and* diastolic blood pressure percentile below the 95th percentile.
*The novel Danish reference values were used as an internal reference in this analysis.
International reference values were based the auscultatory measurements of office blood pressure in the upright sitting position.
The novel Danish reference values were based on blood pressure measurements in the supine position, arm supported, using Welch Allyn Pro-BP-3400 electronic device (Welch Allyn, New York).

Table S8: Difference in blood pressure (mmHg) reference value at the 95th percentile between the novel Danish model (oscillometric) and international guidelines (auscultatory)

	GIRLS				BOYS			
	Systolic (mmHg)		Diastolic (mmHg)		Systolic (mmHg)		Diastolic (mmHg)	
Age (years)	**ESH**	**AAP/ESC**	**ESH**	**AAP/ESC**	**ESH**	**AAP/ESC**	**ESH**	**AAP/ESC**
4	-1	-2	0	+1	-2	+1	0	+3
5	-2	-2	-1	0	-2	+1	-3	0
6	-1	-1	-3	-1	-3	0	-4	-1
7	-1	0	-3	-1	-2	+1	-6	-3
8	-2	0	-4	-2	-2	0	-7	-3
9	-2	-1	-4	-2	-2	+1	-8	-5
10	-2	0	-5	-3	-1	+2	-8	-5
11	-3	0	-5	-3	-3	+1	-9	-6
12	-3	-2	-6	-4	-2	0	-8	-5
13	-3	-3	-6	-4	-4	-3	-8	-5
14	-5	-2	-7	-5	-4	-6	-8	-7
15	-3	-2	-7	-5	-5	-6	-7	-9

The difference (mmHg) is calculated as the Danish reference value minus the reference value from the ESH guideline (original American dataset) or the AAP/ESC guidelines (weight-modified American dataset). A negative value shows that the Danish reference value at the 95th percentile is lower than the reference value in the corresponding guideline for a given sex and age at the 50th height percentile.
ESH: European Society of Hypertension; AAP: American Academy of Pediatrics; ESC: European Society of Cardiology.
International reference values were based the auscultatory measurements of office blood pressure in the upright sitting position.
The novel Danish reference values were based on blood pressure measurements in the supine position, arm supported, using Welch Allyn Pro-BP-3400 electronic device (Welch Allyn, New York).

Figure S3: Comparison of Danish percentiles (oscillometric) and American percentiles (auscultatory) for systolic and diastolic blood pressure

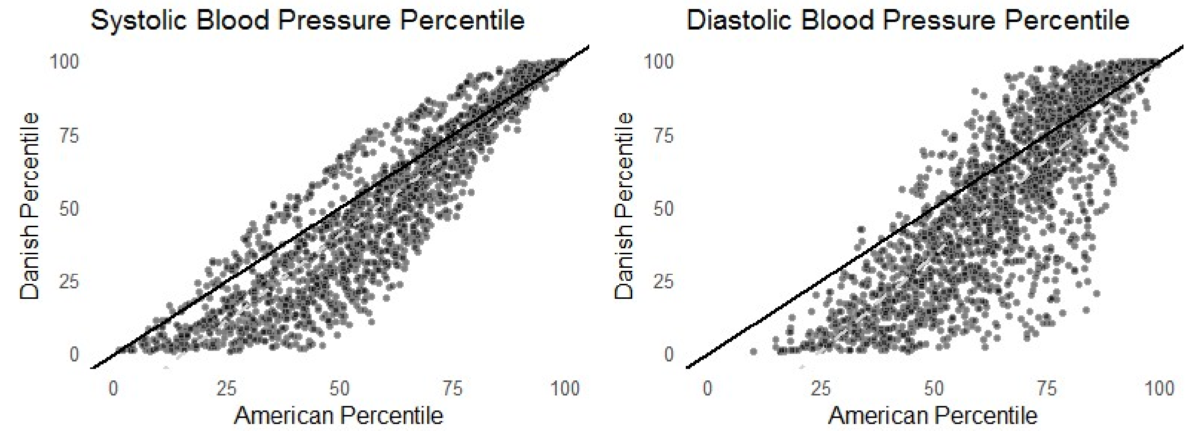

A: Correlation plot. Comparison of blood pressure percentiles in LOFUS when applying the Danish Model vs. the American model. Solid line: line of equality. Dashed line: linear regression line (R^2=0.84).

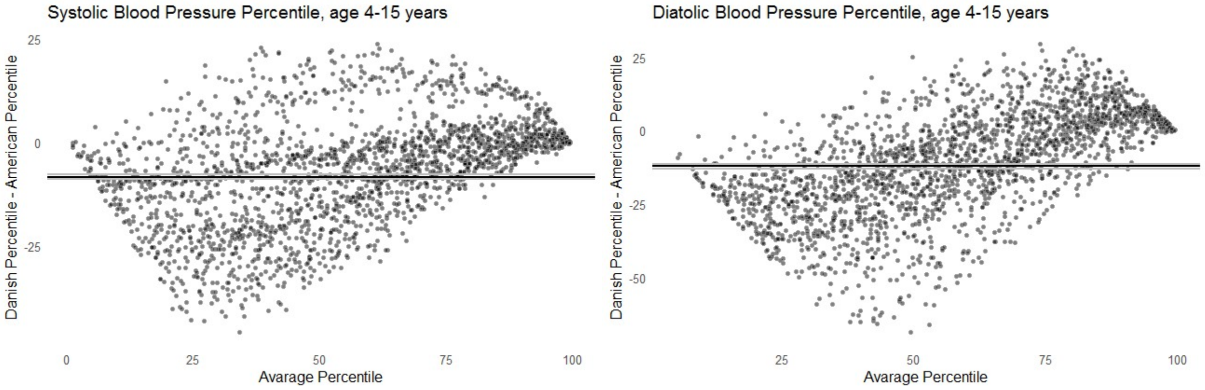

B: Bland-Altman plot (absolute). Black line: mean difference. Grey line: 95% CI intervals of mean difference.

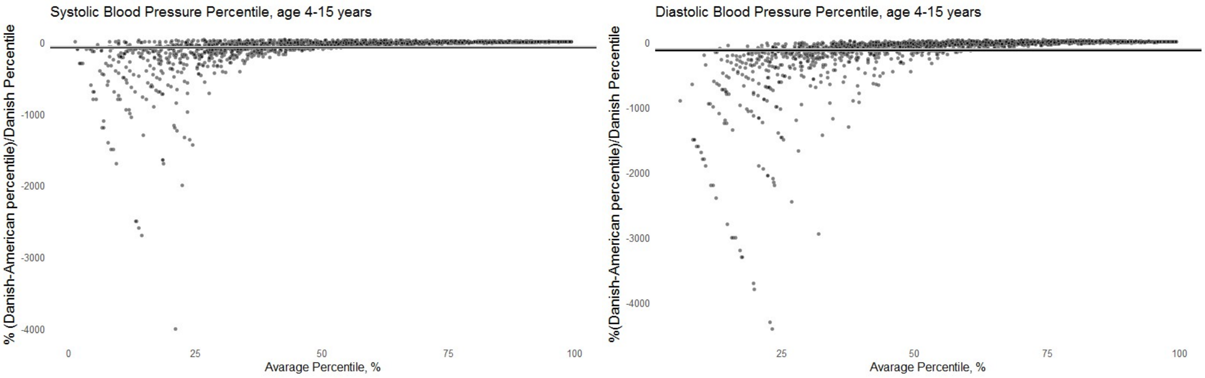

C: Bland-Altman plot (relative). Mean percentage difference (systolic): -79, 95%CI(-68 - -89). Mean percentage difference(diastolic): -130, 95%CI (-113 - -147).
